# Social network analysis to predict social behavior in dairy cattle

**DOI:** 10.3168/jdsc.2023-0507

**Published:** 2024-04-20

**Authors:** H. Marina, W.F. Fikse, L. Rönnegård

**Affiliations:** 1 Department of Animal Biosciences, Swedish University of Agricultural Sciences, SE-750 07 Uppsala, Sweden; 2 Växa, Swedish University of Agricultural Sciences, SE-756 51 Uppsala, Sweden; 3 School of Information and Engineering, Dalarna University, SE-791 88 Falun, Sweden; 4 The Beijer Laboratory for Animal Science, Swedish University of Agricultural Sciences, SE-750 07 Uppsala, Sweden

## Abstract

Dairy cattle are frequently housed in freestalls with limited space, affecting social interactions between individuals. Social behavior in dairy cattle is gaining recognition as a valuable tool for identifying sick animals, but its application is hampered by the complexities of analyzing social interactions in intensive housing systems. In this context, precision livestock technologies present the opportunity to continuously monitor dyadic spatial associations on dairy farms. The aim of this study is to evaluate the accuracy of predicting social behavior of dairy cows using social network analysis. Daily social networks were built using the position data from 149 cows over 14 consecutive days of the study period. We applied the separable temporal exponential random graph models to estimate the likelihood of formation and persistence of social contacts between dairy cows individually and to predict the social network on the subsequent day. The correlation between the individual degree centrality values, the number of established social contacts per individual, between the predicted and observed networks ranged from 0.22 to 0.49 when the structural information from network triangles was included in the model. This study presents a novel approach for predicting animal social behavior in intensive housing systems using spatial association information obtained from a real-time location system. The results indicate the potential of this approach as a crucial step toward the larger goal of identifying disruptions in dairy cows' expected social behavior.

## Highlights

• Social network analysis was applied to predict social behavior in dairy cattle.
• Structural network information improved the accuracy of predicting social behavior.
• Degree centrality correlations showed values ranging from 0.22 to 0.49.
• We introduced a novel approach to predict social behavior in dairy cattle.

## Graphical Abstract

**Figure:**
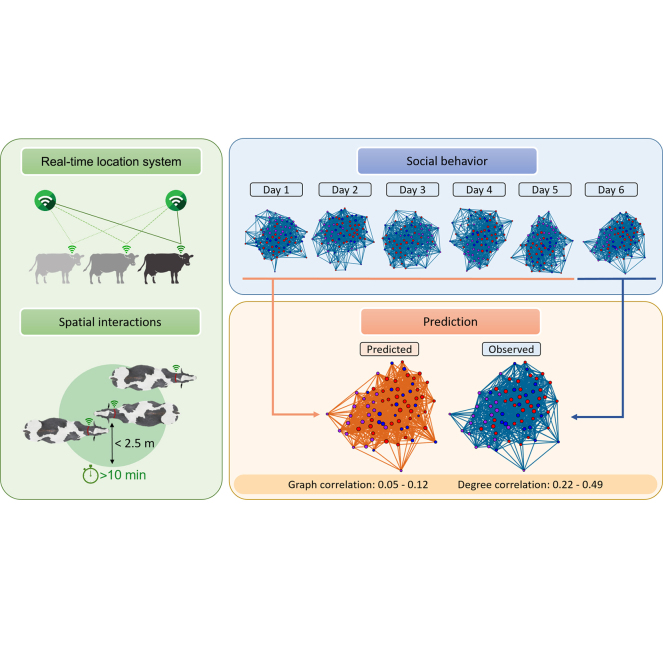
**Summary:** This study evaluated the accuracy of predicting social behavior in dairy cows by applying social network analysis to spatial association data monitored by a real-time location system. Social networks were built based on spatial associations between dairy cattle. Predictive models fitted social network information from 5 consecutive days to anticipate the subsequent day's social network. The accuracy of predicting the number of contacts per individual was higher compared with the accuracy of anticipating the global social network.

Dairy cattle are frequently housed in freestalls with restricted space, leading to inevitable and often limited social interactions between individuals. Dairy cattle can develop meaningful social relationships, and the expression of this innate behavior is paramount to their well-being (Fraser et al., 1997; Beaver et al., 2020). Disruption of social relationships may induce stress, prompting aggressive and anomalous behaviors that may ultimately have long-term health consequences (Grant and Albright, 2001; von Keyserlingk et al., 2008). Social bonding in dairy cattle can play a key role in disease susceptibility (Huzzey et al., 2007; Kappeler et al., 2015). In addition, sick animals often exhibit sickness behavior, which can include a decrease in general activity, food intake, and social behavior (Dantzer and Kelley, 2007). Consequently, social behavior is becoming an increasingly recognized tool for identifying sick animals (Weary et al., 2009). However, the detection of abnormal social behavior is challenging due to the limited possibilities for animal isolation in intensive housing systems (Proudfoot et al., 2012). In this context, precision livestock farming technologies, such as real-time location systems (**RTLS**), allow continuous monitoring of dyadic spatial associations on dairy farms. The utilization of RTLS information provides valuable insights into the social contacts of dairy cattle, even within the confines of intensive housing systems. Social network analysis has been described as an appropriate tool for understanding in detail the dynamics of dyadic social interaction information provided by these systems (Wey et al., 2008). Using this approach, previous studies have described a short-term stability (i.e., 2 wk) of the role of dairy cows within the social networks (Hansson et al., 2023; Marina et al., 2024).

The aim of this study was to assess the accuracy of predicting social behavior in dairy cows by applying social network analysis utilizing RTLS information. Anticipating social behavior may allow the detection of abnormal animal behavior and the causes related to it. The authors declare that according to the Swedish Animal Welfare Act, no ethical approval is needed for this type of study.

This study was conducted on a commercial noninsulated freestall farm that houses around 210 dairy cows (Figure 1). The barn is divided into 2 different management groups. Cows were routinely switched between groups at approximately 170 DIM; hence, these 2 lactation groups are referred to as the early- and late-lactation groups, respectively. The dairy cows were milked twice a day in a 2 × 12 milking parlor from GEA Farm Technologies. Further information on farm management characteristics can be found in Hansson et al. (2023). Individual cow information on age and pedigree was extracted from the Swedish official milk recording scheme, whereas information about parity was provided by the farm. The parity variable was categorized into 3 classes (1, 2, and 3+). Of the total number of lactating dairy cows housed on this farm, only 149 cows that were present in one of the lactation groups for the entire studied period and had information on all characteristics (73 cows in the early lactation group and 76 cows in the late lactation group) were included in the analyses. The lactating cows were equipped with a tag mounted on their collars, which allowed the automatic collection of position data with a 1-s fix rate using an ultra-wideband RTLS (CowView, GEA Farm Technologies), with an accuracy of 0.78 m (Hansson et al., 2023). The present study includes 14 d of positioning data (from October 16 to 29, 2020). During this period, data missing averaged 33.34% (∼8 h/d), with the most common scenario being a single second missing. Missing position information was interpolated by applying the modified Akima interpolation (Akima, 1970) implemented in MATLAB (MATLAB, 2020) following Ren et al. (2022). All missing positions were filled after the interpolation procedure. R statistical software version 4.2.0 (R Core Team, 2022) was used for subsequent analyses. The source code used is publicly available at https://github.com/CSI-DT/SNA.

**Figure 1:**
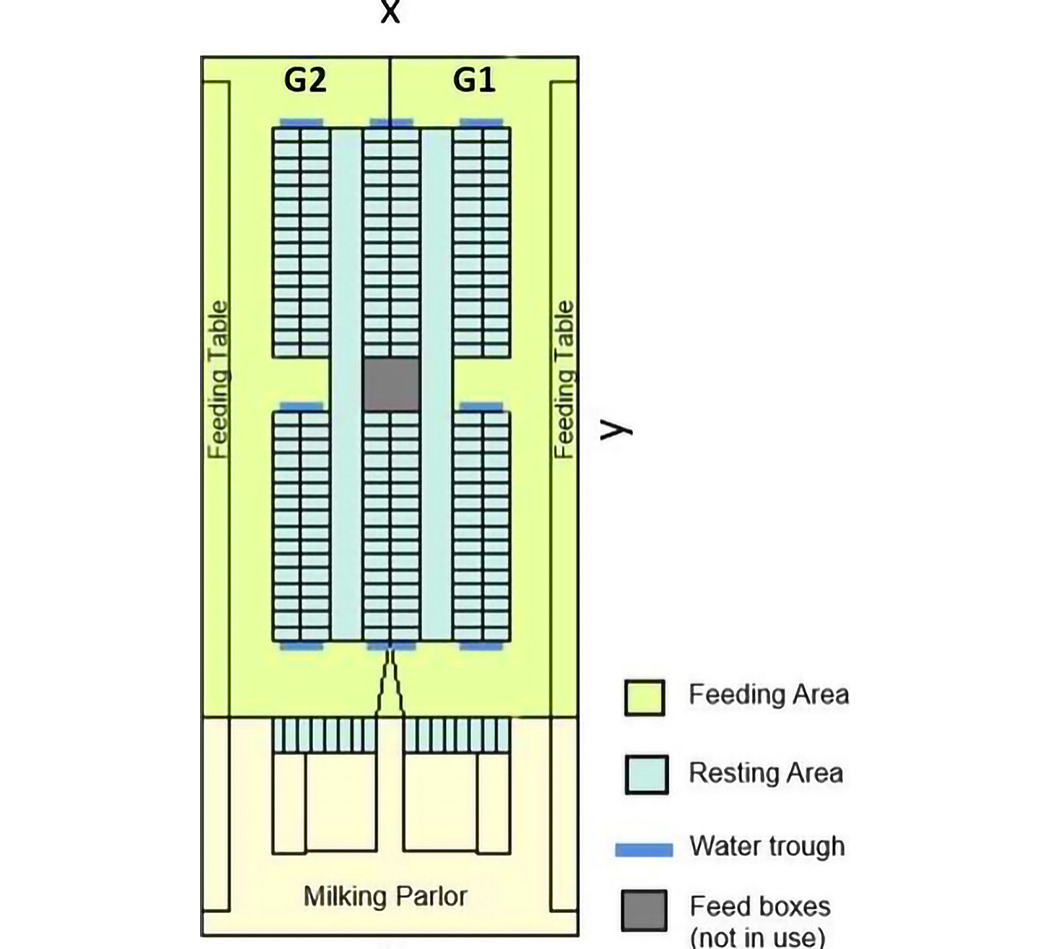
Schematic map of the freestall barn (74 × 33 m) divided into 2 lactation groups: early (G1) and late (G2) lactation, where y and x represent the direction of the y-axis and x-axis, respectively. The cubicles are in the center of the barn, and the feeding tables are on either side. The beige area is outside the boundaries of the cows in the milking group, except for transport between the pen area and the milking parlor.

The barn area was divided into 2 functional areas: the resting area, consisting of the cubicles in the center of the barn and the inner alleys, and the feeding area, consisting of the feeding tables and outer alleys. The time each cow spent with the rest of the herd within a 2.5 m radius was quantified per functional area using position data. The justification for this distance threshold is derived from the average cubicle width of 1.25 m in the resting area; thus, the maximum distance between 2 cows lying in consecutive cubicles would be approximately 2.5 m. To avoid stochastic contacts, we filtered out daily contacts between cows of less than 600 s (10 min). Previous studies conducted by our research group have described no effects of varying the time and distance thresholds on the results obtained from different models (Hansson et al., 2023; Marina et al., 2024). Affiliative social interactions were found positively correlated with prolonged proximity contacts in dairy cows and calves, whereas short encounters were associated with agonistic interactions only in calves (Boyland et al., 2016; Ben Meir et al., 2023). This supports the use of this method for measuring social contacts in dairy cattle. Daily social networks were constructed using position information from 149 cows selected over 14 consecutive days of the study period via the SNA package (Butts, 2008). The pedigree information concerning the 149 cows comprised a total of 8,594 animals over 22 generations. This information was used to estimate the additive relationship matrix. Age information was used to estimate the kindergarten effect following Marina et al. (2024). The kindergarten effect is a binary variable, having the value 1 if both cows were born 7 d apart on the same farm, and 0 otherwise.

Social networks were visualized as sociograms using the igraph package (Csárdi and Nepusz, 2006), with nodes representing individual cows, and spatial contacts between cows represented in a binary format. Further, to determine the optimal number of days to consider in models predicting social behavior, we estimated the pairwise graph correlation between the daily social networks within each functional area and lactation group (e.g., 1–2, 1–3, …, 13–14). Graph correlation determines the similarity between 2 network graphs by dividing the covariance of the adjacency matrices by their SD. The resulting statistic can be interpreted as a standard Pearson's product-moment correlation coefficient (Gjesfjeld, 2015). The dynamic social networks were modeled using separable temporal exponential random graph models (**STERGM**) described by Krivitsky and Handcock (2014). The STERGM approach models the probability of a new spatial contact given that there was no contact on the previous day (formation) and the probability of a spatial contact given that it was established on the previous day (persistence). Formation and persistence events were modeled including fixed effects of parity of both cows in the dyad, additive genetic relationship between both cows (estimated using pedigree information), and the kindergarten effect. The homophily effects of parity, kindergarten effect, and kinship relationships on the likelihood of formation and persistence of social contacts in dairy cattle have been described by Marina et al. (2024). In network analysis, variables representing information external to the network are classified as exogenous variables (e.g., cow information), whereas endogenous variables refer to factors determined by the structure of the network itself (e.g., number of triangles in the network).

In this study, the model was fitted incorporating these exogenous variables (**M1**), and further enhancing it by integrating the structural information of network triangles (**M2**). A triangle consists of 3 nodes, each of which is connected to the other 2 nodes. Incorporating triangles into network analysis can assist in understanding important structural properties of networks (Durak et al., 2012). Further explanations about the STERGM method can be found in Marina et al. (2024). The model was fitted separately for each functional area and lactation group using sliding windows of 5 consecutive days. The utilization of a 5-d window length was determined by the decreasing trend in the graph correlation between the social networks over time, as explained later. Moreover, window lengths below 5 d led to stronger convergence problems in the M2. The STERGM estimates were used to predict the animals' social behavior on the subsequent day. Social contacts were predicted individually based on the likelihood of occurrence of each dyad, derived from the estimates of formation and persistence calculated by STERGM and the information on the previous network structure. A total of 100 networks were generated per prediction scenario. In addition, to compare these results with a naïve model, the animal identifications of the consecutive networks were randomly permuted 100 times.

The accuracy of network predictions was assessed by correlation analysis, including graph correlation and degree centrality correlation with the observed network. On the one hand, the graph correlation offers valuable insights into the accuracy of predicting the specific social contacts between dairy cows within the herd. On the other hand, the Spearman correlation of the degree centrality values examines the total number of social contacts established by individual nodes. Gjesfjeld (2015) described how 3 common centrality parameters (degree, betweenness, and eigenvector) vary when different nodes were excluded from the network. Degree exhibited robust stability when specific individuals were removed from the network. Commercial dairy farms usually group cows by stage of lactation, which leads to regrouping of animals (Grant and Albright, 2001; Hu et al., 2023). Hence, degree centrality is a suitable parameter to generate insights into the accuracy of predicting the social roles of dairy cows.

The pairwise graph correlation between the daily social networks revealed that as the temporal distance between the compared days increased, the corresponding graph correlation values decreased (Figure 2). This decrease in graph correlation values over time supports a progression in the dynamics of the social networks. Based on these observations, sliding windows of 5 consecutive days were fitted in the prediction model. The higher similarity observed between consecutive social networks suggests the potential for short-term prediction of social network structure and individual social behavior.

**Figure 2:**
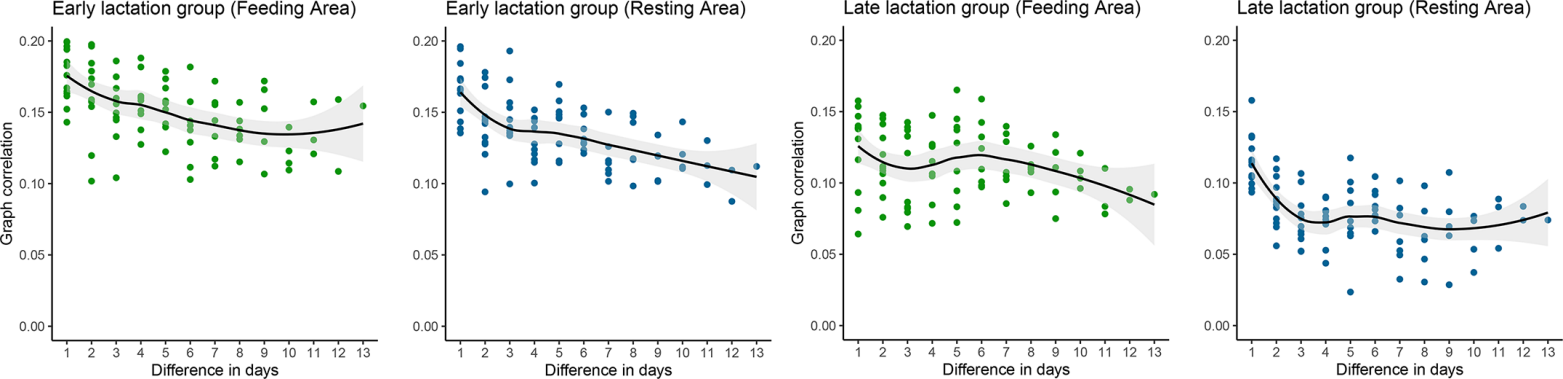
Correlograms of the graph correlation results per lactation group and per functional area (feeding [in green] and resting [in blue]). Each plot includes a locally fitted polynomial regression curve (smoothed line), encapsulated by a shaded region representing the 95% CI.

The utilization of 5-d sliding windows for prediction resulted in 9 predicted scenarios for each lactation group and functional area. Out of the 36 prediction scenarios fitted per model, all converged successfully for M1, whereas 11 scenarios failed to converge for M2. In the early lactation group, 7 scenarios did not converge for the resting area. In the late-lactation group, 2 scenarios did not converge for each functional area. Only models that converged were compared in this study. The reduced number of days considered in the model could have led to model degeneracy when triadic terms (triangles) are included in the model, particularly within networks characterized by high levels of structural heterogeneity (e.g., dense regions with many triangles or high-degree nodes mixed with low-density regions; O'Malley and Marsden, 2008). Such structural complexity may impede model convergence. To address potential degeneracy, one approach is to increase either the number of days or individuals considered in the analysis. However, increasing the number of days considered in the model could compromise the accuracy of the prediction as it may not reflect the dynamic nature of social relationships in cattle.

The STERGM estimates and the prior network state were used to generate 100 social networks per prediction scenario. Across all converged scenarios, the average graph correlation values ranged from 0.01 to 0.12, accompanied by SD from 0.01 to 0.02. Recall that the graph correlations between social networks from consecutive days were lower than 0.20 (Figure 2). Similarly, degree centrality correlation values spanned from 0.06 to 0.49, with corresponding SD varying from 0.06 to 0.12. In addition, the accuracy of the betweenness and eigenvector centrality parameters was also examined, giving very similar results to those obtained for degree centrality (data not shown). Moreover, the average values reported by the naïve model for graph correlation and degree centrality correlation were 0.00 and −0.01, with SD of 0.02 and 0.12, respectively. The difference in accuracy results between graph correlation and degree centrality correlations is because the graph correlation evaluates the accuracy of predicting specific dyads within the network, whereas degree centrality only quantifies the prediction of the total number of social contacts. Although a superior predictive accuracy was achieved for degree centrality, it is noteworthy that cows form preferential relationships with similar and familiar individuals (Vázquez-Diosdado et al., 2023; Marina et al., 2024). Hence, the use of specific dyads information in combination with the total number of social contacts emerges as the most promising approach to detect disruptions in social behavior.

Across all converged scenarios, M2 achieved superior performance, with higher graph and degree centrality correlations and lower SD in accuracy results compared with M1 (Figure 3). Including this structural information substantially increased the degree centrality correlation values, with results ranging from 0.22 to 0.49 (Figure 3). These outcomes highlight the importance of considering the structural information of network triangles for predicting social behavior in dairy cattle. Triangles are a fundamental element of social networks, and including information about them can provide a more complete picture of the social network structure (Durak et al., 2012). The inclusion of this term in the model implicates an assumption of dependency between edges in the network. Consequently, unobserved interactions (i.e., caused by untagged animals or observational errors) could affect the interpretation of results and the accuracy of predictions (James et al., 2009). Nevertheless, the results of the models fitted in this study were not affected by variations in the number of individuals considered in the social networks (data not shown).

**Figure 3:**
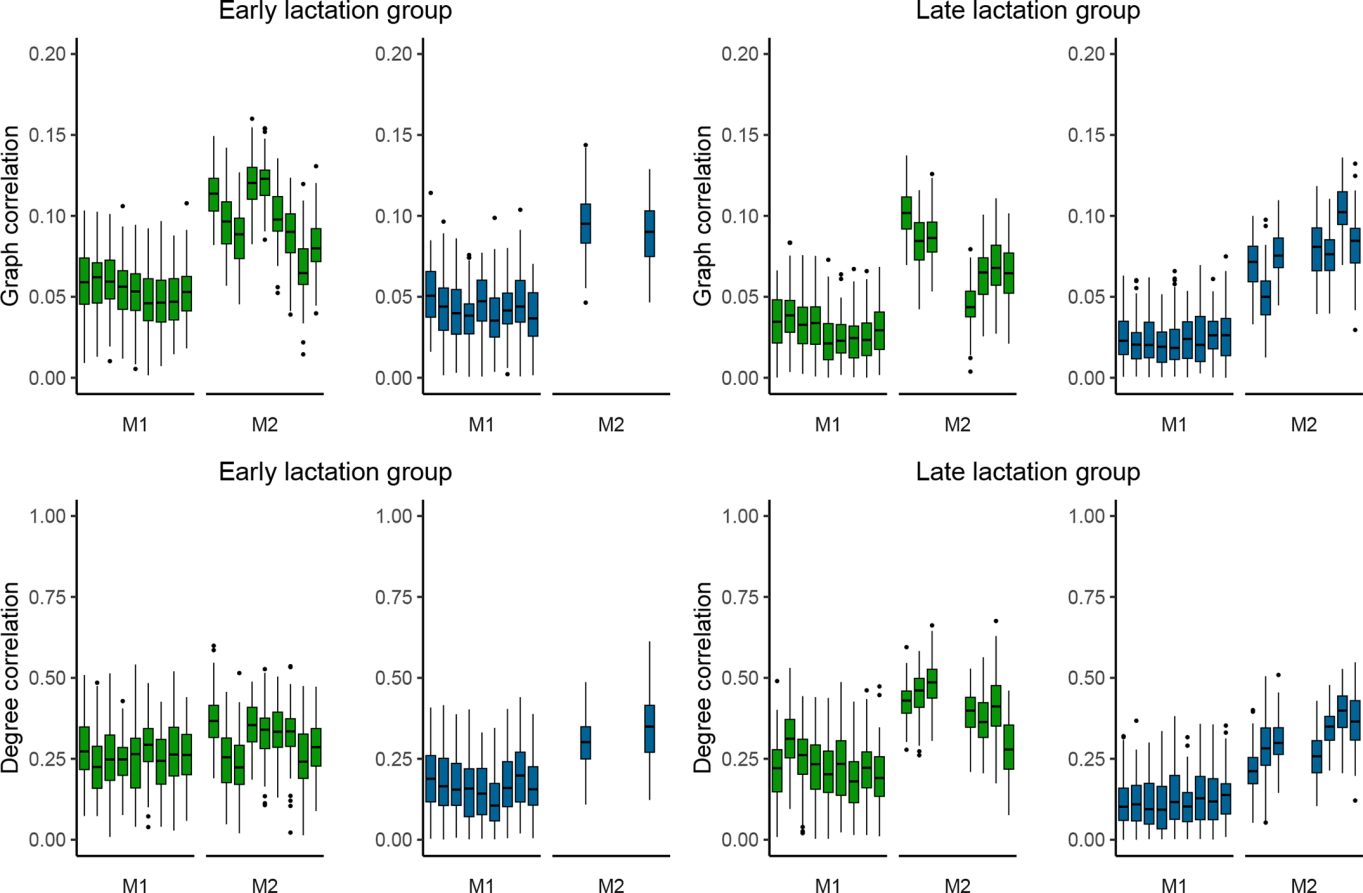
Box plots represent the graph correlations and degree correlations between the predicted and observed networks per prediction scenario, lactation group, and functional area (feeding [in green] and resting [in blue]). Upper and lower edges of boxes represent medians (solid line) ±25th percentile range. Whiskers denote 10th–90th percentiles, and dots denote outliers. The accuracy of the predictions is shown for both models: M1 (exogenous variables) and M2 (exogenous variables and structural information of the network triangles).

Further studies are encouraged to focus on improving the predictive accuracy of the social networks in dairy cattle. This could involve including structural network information other than triangles, or by considering contact duration instead of analyzing binary social networks. Other factors that may affect the accuracy of predictions include regrouping events and the number of herds considered. On the one hand, regrouping events can disrupt the stability of social networks, thereby affecting the accuracy of estimates (Rocha et al., 2020). On the other hand, considering a larger number of herds could provide more accurate results from the model.

The use of social behavior as an early indicator for identifying sick animals is limited by the complexity of analyzing this information in intensive housing systems (Proudfoot et al., 2012). However, sickness behavior can affect general activity levels, feeding patterns, and social interactions (Dantzer and Kelley, 2007), all of which could influence spatial associations measured in this study and, consequently, the social network structure. This study presents a novel approach using RTLS-monitored spatial association data for predicting animal social behavior in intensive housing systems. Future research should prioritize comparative analysis of accuracy when integrating information from different window lengths in populations of different sizes.

## References

[bib1] Akima H. (1970). A new method of interpolation and smooth curve fitting based on local procedures. J. Assoc. Comput. Mach..

[bib2] Beaver A., Proudfoot K.L., von Keyserlingk M.A.G. (2020). Symposium review: Considerations for the future of dairy cattle housing: An animal welfare perspective. J. Dairy Sci..

[bib3] Ben Meir Y.A., Garcia F., Cohen-Zinder M., Shabtay A. (2023). Use of proximity loggers to estimate affiliative and agonistic relationships among group-housed Holstein calves. J. Appl. Anim. Welf. Sci..

[bib4] Boyland N.K., Mlynski D.T., James R., Brent L.J.N., Croft D.P. (2016). The social network structure of a dynamic group of dairy cows: From individual to group level patterns. Appl. Anim. Behav. Sci..

[bib5] Butts C.T. (2008). Social network analysis with sna. J. Stat. Softw..

[bib6] Csárdi G., Nepusz T. (2006). The igraph software package for complex network research. InterJournal Complex Sy.

[bib7] Dantzer R., Kelley K.W. (2007). Twenty years of research on cytokine-induced sickness behavior. Brain Behav. Immun..

[bib8] Durak N., Pinar A., Kolda T.G., Seshadhri C. (2012). Proceedings of the 21st ACM International Conference on Information and Knowledge Management.

[bib9] Fraser D., Weary D.M., Pajor E.A., Milligan B.N. (1997). A scientific conception of animal welfare that reflects ethical concerns. Anim. Welf..

[bib10] Gjesfjeld E. (2015). Network analysis of archaeological data from hunter-gatherers: Methodological problems and potential solutions. J. Archaeol. Method Theory.

[bib11] Grant R.J., Albright J.L. (2001). Effect of animal grouping on feeding behavior and intake of dairy cattle. J. Dairy Sci..

[bib12] Hansson I., Silvera A., Ren K., Woudstra S., Skarin A., Fikse W.F., Nielsen P.P., Rönnegård L. (2023). Cow characteristics associated with the variation in number of contacts between dairy cows. J. Dairy Sci..

[bib13] Hu T., Zhang J., Zhang X., Chen Y., Zhang R., Guo K. (2023). The development of smart dairy farm system and its application in nutritional grouping and mastitis prediction. Animals (Basel).

[bib14] Huzzey J.M., Veira D.M., Weary D.M., Von Keyserlingk M.A.G. (2007). Prepartum behavior and dry matter intake identify dairy cows at risk for metritis. J. Dairy Sci..

[bib15] James R., Croft D.P., Krause J. (2009). Potential banana skins in animal social network analysis. Behav. Ecol. Sociobiol..

[bib16] Kappeler P.M., Cremer S., Nunn C.L. (2015). Sociality and health: Impacts of sociality on disease susceptibility and transmission in animal and human societies. Philos. Trans. R. Soc. Lond. B Biol. Sci..

[bib17] Krivitsky P.N., Handcock M.S. (2014). A separable model for dynamic networks. J. R. Stat. Soc. Series B Stat. Methodol..

[bib18] Marina H., Ren K., Hansson I., Fikse F., Nielsen P.P., Rönnegård L. (2024). New insight into social relationships in dairy cows and how time of birth, parity, and relatedness affect spatial interactions later in life. J. Dairy Sci..

[bib19] MATLAB (2020).

[bib20] O'Malley A.J., Marsden P.V. (2008). The analysis of social networks. Health Serv. Outcomes Res. Methodol..

[bib21] Proudfoot K.L., Weary D.M., von Keyserlingk M.A.G. (2012). Linking the social environment to illness in farm animals. Appl. Anim. Behav. Sci..

[bib22] R Core Team (2022).

[bib23] Ren K., Alam M., Nielsen P.P., Gussmann M., Rönnegård L. (2022). Interpolation methods to improve data quality of indoor positioning data for dairy cattle. Front. Anim. Sci..

[bib24] Rocha L.E.C., Terenius O., Veissier I., Meunier B., Nielsen P.P. (2020). Persistence of sociality in group dynamics of dairy cattle. Appl. Anim. Behav. Sci..

[bib25] Vázquez-Diosdado J.A., Occhiuto F., Carslake C., Kaler J. (2023). Familiarity, age, weaning and health status impact social proximity networks in dairy calves. Sci. Rep..

[bib26] von Keyserlingk M.A.G., Olenick D., Weary D.M. (2008). Acute behavioral effects of regrouping dairy cows. J. Dairy Sci..

[bib27] Weary D.M., Huzzey J.M., Von Keyserlingk M.A.G. (2009). Board-invited review: Using behavior to predict and identify ill health in animals. J. Anim. Sci..

[bib28] Wey T., Blumstein D.T., Shen W., Jordán F. (2008). Social network analysis of animal behaviour: A promising tool for the study of sociality. Anim. Behav..

